# Mitochondrial Epigenetics: Non-Coding RNAs as a Novel Layer of Complexity

**DOI:** 10.3390/ijms21051838

**Published:** 2020-03-06

**Authors:** Giovanna C. Cavalcante, Leandro Magalhães, Ândrea Ribeiro-dos-Santos, Amanda F. Vidal

**Affiliations:** 1 Laboratory of Human and Medical Genetics, Federal University of Pará, Av. Augusto Correa, 01, 66075-970 Belém, PA, Brazil; giovannaccavalcante@gmail.com (G.C.C.); leandromag@me.com (L.M.); akelyufpa@gmail.com (Â.R.-d.-S.); 2 Graduate Program in Genetics and Molecular Biology, Federal University of Pará, Av. Augusto Correa, 01, 66075-110 Belém, PA, Brazil; 3 Graduate Program in Oncology and Medical Sciences, Center of Oncology Researches, Federal University of Pará, Rua dos Mundurucus, 4487, 66073-005 Belém, PA, Brazil

**Keywords:** mitochondria, epigenetics, non-coding RNAs

## Abstract

Mitochondria are organelles responsible for several functions involved in cellular balance, including energy generation and apoptosis. For decades now, it has been well-known that mitochondria have their own genetic material (mitochondrial DNA), which is different from nuclear DNA in many ways. More recently, studies indicated that, much like nuclear DNA, mitochondrial DNA is regulated by epigenetic factors, particularly DNA methylation and non-coding RNAs (ncRNAs). This field is now called mitoepigenetics. Additionally, it has also been established that nucleus and mitochondria are constantly communicating to each other to regulate different cellular pathways. However, little is known about the mechanisms underlying mitoepigenetics and nuclei–mitochondria communication, and also about the involvement of the ncRNAs in mitochondrial functions and related diseases. In this context, this review presents the state-of-the-art knowledge, focusing on ncRNAs as new players in mitoepigenetic regulation and discussing future perspectives of these fields.

## 1. Introduction

Mitochondria are cytoplasmic organelles that play a central role in energy generation by oxidative phosphorylation (OXPHOS) and in several other mechanisms involved in cellular homeostasis [1]. These organelles are commonly called “powerhouses of the cell” because of their importance.

In the 1960s, Lynn Margulis (then Lynn Sagan) proposed that mitochondria were originated from free-living cells (called “protomitochondria”) that were ingested into the cytoplasm of anaerobes, establishing a mandatory endosymbiosis. This would be the first step to the origin of eukaryotes from prokaryotes in an aerobic environment [2]. Later on, it has been suggested that mitochondria originated from Alphaproteobacteria [3]. Further studies have supported these theories, but many efforts are still being made to understand this evolutionary process and to firmly place mitochondria in the phylogeny involved in it [4].

Due to their origin, mitochondria possess their own genome (mtGenome or mitogenome), which presents individual functions and communicates with nuclear genome. Recently, it has been shown that mitochondrial DNA (mtDNA), like nuclear DNA (nDNA), is regulated by epigenetic mechanisms. This regulation is called mitoepigenetics [5].

Whether by genetic variants or epigenetic control, alterations in the mtGenome leading to mitochondrial dysfunction may influence the development of different diseases. Thus, it is crucial to understand these mechanisms.

In this review, we discuss the complexity of mitochondrial genetics and epigenetics, highlighting the role of non-coding RNAs (ncRNAs) inside this organelle, mainly in mammals. We also discuss the possible impact of epigenetic regulation in human mitochondrial diseases and future perspectives in this scenario.

## 2. Mitochondrial Functions

As previously mentioned, mitochondria are responsible for many different functions in cellular homeostasis, but these organelles are mainly known for aerobic energy generation, especially by OXPHOS, that occurs in the mitochondrial cristae (inner membrane’s convolutions) through five protein complexes (I-V).

During OXPHOS, these complexes work along with electron carriers as an electron transport chain (ETC), also known as respiratory chain, receiving and donating electrons to the next complex until reaching complex V [6].

In brief, electrons may enter the ETC mainly via complex I (by oxidation of NADH and reduction of ubiquinone) or alternatively via complex II (by oxidation of succinate and reduction of ubiquinone); regardless, electrons are transferred to complex III by carrier ubiquinol and then to complex IV by carrier cytochrome c, which will transfer electrons to molecular oxygen [7,8]. It is noteworthy that, in different steps of this process, reactive oxygen species (ROS) are generated.

As these electrons are passed from one complex to the other (complexes I, III and IV only), protons are pumped out and back into the inner membrane, creating an electrochemical gradient that will be the energy source to complex V (or ATP synthase), which converts adenosine diphosphate (ADP) to adenosine triphosphate (ATP) by adding a phosphate group to it, completing OXPHOS [6].

Krebs cycle (also known as citric acid cycle or tricarboxylic acid cycle), another energy generation process, also takes place in mitochondria, but in the mitochondrial matrix. This mechanism results in 2 ATP, 6 NADH, 2 FADH_2_, 6 H^+^ and 4 CO_2_ molecules per glucose molecule, of which NADH and FADH_2_ will then supply ETC with electrons [7]. After all processes of the aerobic cellular respiration (glycolysis in the cytosol and Krebs cycle and OXPHOS in the mitochondria), one glucose molecule will have generated up to 38 ATP molecules, most of which are generated inside the mitochondria.

Mitochondria are also highlighted by their major roles in apoptosis, a type of Regulated Cell Death (RCD) class. In addition to providing energy for the whole mechanism, mitochondrial outer membrane permeabilization (MOMP) is a key step in the apoptotic intrinsic pathway (or mitochondrial pathway). MOMP is considered to be a “point of no return” in apoptosis [9]. A recent review by our research group has thoroughly discussed the mitochondrial role and interactions with nuclear proteins in the apoptotic process [10].

Taking into account the importance of this organelle, dysregulation of mitochondrial functions may lead to cellular imbalance and damage, which might culminate in different diseases, including cancers and neurodegenerative conditions [11,12,13,14,15]. Such dysregulation might be due to genetic, epigenetic or environmental factors. In this context, it is notable that genetic dysfunction of mitochondria could be dependent on alterations in both nuclear and mitochondrial genomes, due to nuclei–mitochondria communication.

## 3. Nuclei–Mitochondria Communication

An intense and constant crosstalk between mitochondria and nucleus is required for cellular homeostasis, in which nucleus controls mitochondrial gene expression and posttranslational modifications (anterograde signaling) and mitochondria control nuclear gene expression and protein activity through signal transport from mitochondria to cytosol (retrograde signaling). This nuclei–mitochondria communication is mediated by a range of proteins and ncRNAs [16,17].

There are several nuclear-encoded transcription factors working together in anterograde regulation, such as mitochondrial RNA polymerase (POLRMT), transcription and mtDNA maintenance factor (TFAM), transcription specificity factors (TFB1M and TFB2M) and transcription termination factor (mTERF) [18]. In addition, there are many nuclear receptors and cofactors connected in anterograde signaling. Among these, PGC-1α is an important cofactor to mitochondrial biogenesis, which in turn is one of the different ways anterograde signaling works to overcome stress and maintain mitochondrial balance [19,20].

According to Weinberg et al. [17], retrograde signaling has also been associated to several pathways, including induction of inflammation and immune response to pathogens through activation of different molecules (e.g., macrophages, T cells, IL-1B), activation of the transcription factor hypoxia inducible factor 1 (HIF-1) and metabolic adaptation through ROS production. In addition, ROS has been described as one of the main retrograde signals in response to environmental stress in plants [21].

Moreover, sirtuins (SIRT) are a protein family responsible for regulation of different metabolic pathways. In mammals, there are seven sirtuins (SIRT1-7), of which three (SIRT3-5) are located in mitochondria and participate in many of the mitochondrial functions (e.g., energy production and apoptosis). Thus, alterations or dysregulation in mitochondrial sirtuins would affect retrograde signaling [22].

As mentioned above, different non-coding RNAs are also involved in anterograde and retrograde signaling. See sections “Epigenetics” and “Non-coding RNAs in mitochondria” for a characterization of ncRNA classes and a more detailed explanation of their impact on mitochondrial function. For now, it is important to understand that ncRNAs (e.g., lncRNAs and miRNAs) take part in these regulatory processes. For instance, mitochondria-associated lncRNAs may be either mitochondria-encoded (retrograde signals) or nuclei-encoded and transported to mitochondria (anterograde signals), working in the modulation of mitochondrial metabolism and structure. Due to their importance to mitochondrial function, these lncRNAs have been suggested as potential biomarkers to diagnosis, prognosis, and treatment of different types of cancer [23].

## 4. Mitochondrial Genome

**Figure 1:**
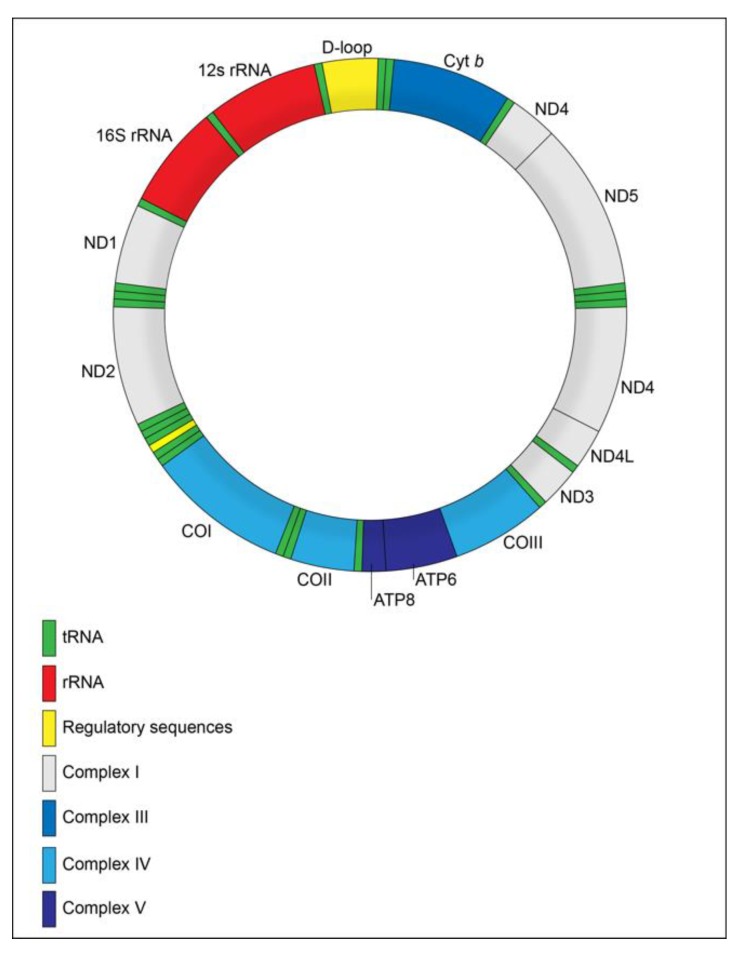
Mitochondrial genome. Map of the 16.6 kb, circular, double-stranded human mitochondrial DNA (mtDNA) molecule, showing the regulatory sequences (in yellow), 22 tRNAs (in green), two rRNAs (in red) and the 13 coding genes classified along the protein five complexes.

It is now well-established that the human mtDNA is a circular molecule of 16,569 bp, composed of 37 genes, of which 13 encode proteins, 22 tRNAs and two rRNAs [24] (Figure 1). It also presents non-coding regions, such as displacement loop (D-loop), responsible for controlling mtDNA replication and transcription [25].

It is notable that, in a cell, there are several mitochondria, each one with a great amount of mtDNA molecules, so that the proportion of a certain alteration may vary among these different copies [26]. The state in which different genotypes of a mutation coexist in the mitochondria of the same cell is called heteroplasmy, while the state of genotype equality is called homoplasmy. It is important to consider that heteroplasmy may affect all regions of the mtGenome and that the frequency of such heteroplasmic variants is heterogeneous between different tissues of the same individual [27]. In addition, it is also relevant that the proportion in which a mitochondrial mutation is present may influence the related phenotype and that this phenotype may only appear when a certain threshold level is exceeded, a phenomenon called mitochondrial threshold effect [28].

During mitochondrial evolution and the mandatory endosymbiosis process, some portions of the mtGenome were transferred to the nuclear genome of the host cells, so that current mtGenome is reduced in terms of gene count when compared to other Alphaproteobacteria (α-proteobacteria) [4]. For that reason, mtDNA and nDNA may work together in shared functions. For instance, in OXPHOS, complex II is encoded by the nuclear genome and the other complexes are encoded by both nuclear and mitochondrial genomes [11].

Even though there are similarities between nDNA and mtDNA, these molecules also differ greatly. First, probably due to mitochondrial origin in bacteria, mtDNA is circular and small in comparison to human nDNA (around 3.3 Gb and linear molecule) [29]. Mitochondrial genome is also 10–20 times more susceptible to molecular alterations in comparison to nuclear genome [30]. This is mainly due to the mitochondrial physical proximity to OXPHOS (because accumulation of ROS may lead to mtDNA damage) and less effective repair mechanisms, including the absence of histones [1,31,32]. Indeed, unlike nDNA, mtDNA is not associated with histones, but these molecules are not “naked”: they are present in areas called mt-nucleoids, associated with different proteins that contribute to mtDNA protection and stability. In fact, there are multiple nucleoids per mitochondrion and each nucleoid may present more than one mtDNA molecule [33].

Some other differences between mtDNA and nDNA were highlighted by van Gisbergen et al. (2015) [11]: (i) mtDNA has only maternal inheritance, rare examples of biparental inheritance have been recently suggested and are still a much-debated topic [34]; (ii) mtDNA is almost totally composed by coding sequences (more than 90%), their genes do not present introns and there is little or no non-coding bases between them; (iii) mtDNA replication occurs regardless of cell cycle (relaxed replication), so its restriction depends only on the availability of the necessary replication machinery; and (iv) transcription in the mtGenome starts in one of the two promoters and occurs in both strands.

Nevertheless, as previously mentioned, genetic alterations may influence mitochondrial functions and may occur in different proportions and regions of the mtGenome. However, it is also imperative to acknowledge that epigenetic features may play a great role in such functions. This matter will be discussed throughout this review.

## 5. Epigenetics

The term “epigenetics” was first used by Conrad H. Waddington in 1942 to refer to a whole complex of developmental processes connecting genotype to phenotype [35]. Currently, epigenetics can be defined as heritable changes in the genome resulting in altered gene expression pattern without affecting the DNA sequence [36].

The fine-tuned gene regulation made by epigenetic mechanisms is crucial to cellular identity and homeostasis, and is involved in vital cellular processes, including transcription, replication, and DNA repair [36,37]. These epigenetic mechanisms occur through three deeply associated layers: DNA methylation, histone modifications and non-coding RNAs [38].

DNA methylation and histone modifications are chemical events made by a plethora of “writer” enzymes responsible for adding chemical residues. Both mechanisms are chromatin-related processes that act synergistically to ensure or block the DNA accessibility to the transcription machinery [36,38].

DNA methylation is deposited and maintained by the DNA-methyltransferases family of enzymes (DNMT1, DNMT2, DNMT3A, DNMT3B, and DNMT3L). They catalyze the addition of a methyl group to position 5 of cytosines using *S*-adenosyl-l-methionine (SAM) as a methyl donor. Nuclear DNA cytosine methylation is related not only to transcriptional repression but also to chromosomal stability. In mammals, 5-methylcytosine (5mC) is mainly found in the context of nuclear cytosine- guanine dinucleotides (CpG sites) in promoter regions, leading to gene silencing [36,39].

Histone modifications represent a group of chemical modifications at the *N*-terminal tail of histones involved in switching between transcriptionally active and inactive chromatin, in DNA repair and in histone degradation. These changes take place at a post-translational level and include methylation, acetylation, phosphorylation, ubiquitination, sumoylation, ADP-ribosylation, and biotibylation. Histone modifications occur at specific aminoacids and in various combinations, resulting in a “histone code” able to silence or activate genes [37,38,39].

Non-coding RNAs (ncRNAs) are RNAs that have several regulatory and structural functions rather than serve as a protein template. In humans, they represent 70% of the genome, evidencing their abundance [40]. There are two main classes of ncRNAs: (i) one represented by structural ncRNAs, including transfer and ribosomal RNAs, which are not traditionally considered into the epigenetics context and (ii) one representing several types of regulatory RNAs, and most of them are epigenetic-related. Based on their length, the epigenetic-related regulatory ncRNAs can be grouped into long ncRNAs (>200 nt), which include the linear (lncRNAs) and the circular ones (circRNAs), and the small ncRNAs (<200 nt), which include microRNAs (miRNAs; 17–23 nt), short interfering RNAs (siRNAs; 20–30 nt) and piwi-interacting RNAs (piRNAs; 27–30 nt) [37].

Most of these epigenetic-related regulatory ncRNAs do not have their roles fully understood, but it has been shown that they can interact with DNA, other classes of RNAs and proteins by cis and trans-regulatory mechanisms, impacting in transcriptional, posttranscriptional and posttranslational control of gene expression [37,40]. Among them, miRNAs are probably the most studied, while circRNAs are the most unknown.

## 6. Mitochondrial Epigenetics

**Figure 2:**
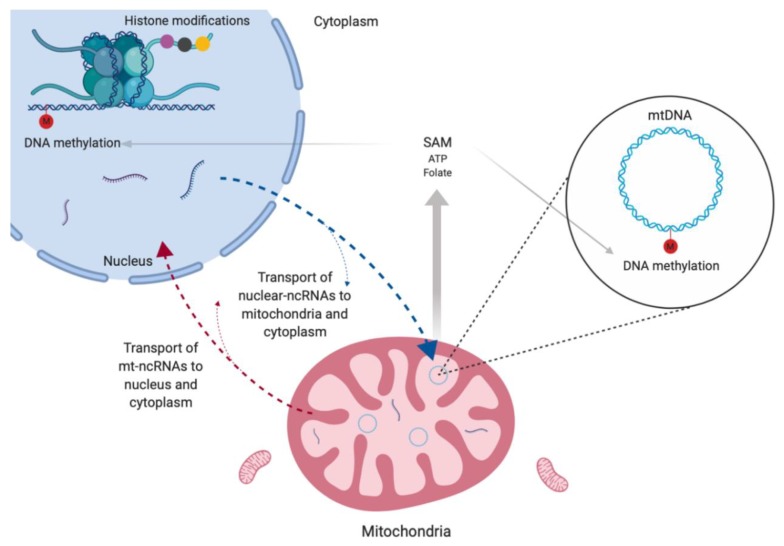
Mitochondrial epigenetics. Scheme showing the occurrence of DNA methylation in mtDNA and of non-coding RNAs (ncRNAs) inside mitochondria. These ncRNAs are encoded by both nucleus and mitochondrial genomes and participate of the nuclei–mitochondria communication. Mitochondrial metabolism is responsible for the production of the universal methyl donor (SAM), which is used in all DNA methylation events in a cell.

Mitochondrial epigenetics is a largely unexplored field, but some studies have demonstrated DNA methylation in mtDNA and the presence of ncRNAs inside mitochondria (Figure 2). Currently, mtDNA methylation is still very controversial; however, evidence suggests that this process is associated not only to transcriptional regulation but also to mtDNA copy number [41,42].

It is important to note that the production of universal methyl donor (SAM) for both nuclear and mitochondrial DNA methylation is regulated by mitochondria metabolism through synthesis of ATP and folate (Figure 2). Actually, most co-substrates required for histone phosphorylation, acetylation and deacetylation are generated through mitochondria [41,42,43]. Thus, these organelles are indirectly related to all DNA methylation and histone modifications events in a given cell.

As mentioned before, although lacking histone packing, mtDNA is not “naked”. In this case, DNA is clustered in TFAM-associated nucleoids (mitochromosome). TFAM is involved in mtDNA packing, replication, transcription and general maintenance of mtDNA. Interestingly, acetylation, phosphorylation, and ubiquitination have been reported for TFAM, but the consequences of these modifications are still unknown. Considering the histone context in nuclei as an epigenetics model of gene regulation, it is possible to assume that these modifications in TFAM alter mitochondrial gene expression [42,44].

It is worth mentioning that several histone family members, especially H2A and H2B, were found within mitochondria by a shotgun proteomics approach. These proteins were detected mainly in the mitochondrial membrane rather than directly bound to DNA and their function in the organelle is not clear [45].

Few studies have demonstrated the presence of ncRNAs inside mitochondria. In humans, this discovery was encouraged mainly by the first human mitochondrial transcriptome in 2011 [46]. Their origin and biological functions are discussed in more details in the next section, but they seem to be crucial to nuclei–mitochondria communication at both anterograde and retrograde signaling levels [41,42].

## 7. Non-Coding RNAs in Mitochondria

Given that mitochondria respond to changes in its membrane potential by alterations in gene expression, it is expected that mtGenome is regulated and expressed in a special manner. In fact, the recent advances in deep sequencing have revealed a complex network of regulation, expression and processing of the mitochondrial transcriptome [46]. In this context, ncRNAs represent one layer of mitochondrial gene regulation complexity.

Interestingly, there are two types of ncRNAs inside mitochondria. The first one is represented by the nuclear-encoded ncRNAs (nuclear-ncRNAs), involved in anterograde communication level and considered a contribution of nuclear RNAs to mitochondrial transcriptome. Studies in different species indicate that there may be several import pathways of these ncRNAs to mitochondria, being most of them ATP-dependent [47]. The second type is the mitochondria-encoded ncRNAs (mt-ncRNAs), which most of them are known as retrograde signals as they could report the mitochondrial activity and state to the host nucleus [23]. In the next subsections, we explore both of types by dividing them according to their classes, summarized in Table 1.

### 7.1. Linear Long Non-coding RNAs

LncRNAs constitute the largest proportion of the nuclear non-coding transcriptome in humans. Several functions have been described in association to them, including transcriptional regulation, protein scaffolding, miRNAs sponging and chromosome remodeling [82]. They are usually classified in six types based on their genomic organization or their relation to protein-coding genes: intronic, intergenic, sense, antisense, bidirectional and enhancer lncRNAs [83].

Overall, the findings suggest that mitochondria-encoded lncRNAs (mt-lncRNAs) have a notable difference in comparison to the nuclear-lncRNAs – some of which are chimeric, and, thus, derived from more than one gene that merge their transcripts as a post-transcriptional product by trans-splicing reactions [48]. It suggests that the mt-lncRNAs could be classified in a different manner from the nuclear ones in order to include their chimerism (e.g., simple mt-lncRNAs and chimeric mt-lncRNAs) [23]. The first evidence of human mitochondrial long ncRNAs was described in 2011 (Table 1). The authors observed that 15% of the mitochondrial transcriptome (after removing rRNA and tRNA) were represented by ncRNAs, including three simple lncRNAs produced by *ND5*, *ND6* and *Cyt b*, and probably regulated by MRPP1, a protein that possesses different functions and is necessary for the accumulation of mitochondrial lncRNAs. Additionally, they found variation in the abundance of these three lncRNAs across 18 different human tissues, indicating their tissue-specificity [49].

In mice, there is a lncRNA that is composed by the 16S mitochondrial RNA plus an inverted repeat of 121 bp of the L strand transcript of the same gene. This mt-lncRNA presented a nuclear localization and was enriched in mice sperm, testis, liver, kidney, brain and spleen [48,50]. Similar results were obtained with human spermatogonia and its overexpression was found in several human proliferating cells, suggesting that this mt-lncRNA is correlated with cell proliferation [51,52].

Later, two similar antisense mitochondrial transcripts containing inverted repeat linked to the 5′ region of the antisense 16S mtRNA (ASncmtRNA-1 and ASncmtRNA-2) were found overexpressed in normal human proliferating cells but not in tumors of different types, suggesting that these transcripts are biomarkers able to distinguish normal from tumor cells [53]. It was shown that the knockdown of these two lncmtRNAs induced apoptosis in several human and mouse tumor cell lines, but not in normal cells, suggesting them as selective targets for a therapy approach against different types of cancer [54,55].

In normal human cells, both ASncmtRNA-1 and ASncmtRNA-2 were found outside mitochondria and especially located in nucleus, associated with heterochromatin and nucleoli, indicating their role in retrograde signaling [56]. In addition, ASncmtRNA-2 might be involved in aging and replicative senescence in endothelial cells, possibly functioning as a non-canonical precursor of miR-4485 and miR-1973 [57].

Human mitochondria also produce similar but sense transcripts that were called SncmtRNA-1 (815-nt inverted repeat) and SncmtRNA-2 (752-nt inverted repeat). The first one was related to cell proliferation in HeLa cells, indicating its potential therapeutic application in cervical cancer. The latter was found only outside mitochondrion and may be involved in the intense crosstalk between this organelle and the host cell. Interestingly, the expression of SncmtRNA-1, SncmtRNA-2, ASncmtRNA-1 and ASncmtRNA-2 is modulated by high risk HPV oncogenes [58]. Strikingly, Yang et al. (2014) found that mitochondrial-encoded lncRNAs constitute the majority of the total cardiac lncRNAs (71%, represented by nine lncRNAs). Their findings demonstrated that the expression of these mt-lncRNAs varies according to the cardiac disease state and it is negatively correlated to nuclear-encoded mitochondrial regulators. This supports the potential of the lncRNAs as biomarkers and indicates its regulation by nuclear factors [59].

There is only limited evidence of nuclear-encoded lncRNAs inside mitochondria. Among these, lncRNA RMRP was found associated with the HuR (human antigen R) and GRSF1 (G-rich RNA sequence-binding factor 1) for accumulation in mitochondrial matrix. This lncRNA is an RNA component of the RNA processing endoribonuclease (RNase MRP), which is responsible for generating primers for mitochondrial DNA replication [60,61].

The steroid receptor RNA activator (SRA) is a nuclear-encoded lncRNA involved in estrogen signaling. This lncRNA is a target of several RPBs including some that are predominantly mitochondrial proteins, such as SLIRP, but the transport mechanism of SRA and SLIRP from the nucleus to mitochondria is still unknown. These findings provided insights into estrogen transduction signaling pathways and offer new targets for therapeutical gain [16,62].

RNase P RNA is a lncRNA subunit of ribonuclease P, which is an enzyme involved in endonucleolytic cleavage of mitochondrial tRNA (mt-tRNA) sequences. Analysis of mitochondrial preparations strongly suggested that the enzyme is located within the organelles, and that the lncRNA is enriched in mitochondrial matrix [46,63]. RNase P RNA, as well as other RNA molecules, are imported into mitochondria by PNPASE (mammalian polynucleotide phosphorylase) [64].

### 7.2. Circular RNAs

Circular RNAs, as their name suggest, are a type of lncRNAs that present a closed circle structure resulted from a covalent bond between its 3′ and 5′ ends. More than 99% of the circRNAs described so far still have their functions unknown, but several roles have been described in association to them, including miRNAs and RBPs sponging, transcriptional regulation, protein templates, and immune system modulators [84,85,86].

In eukaryotes, nuclear-encoded circRNAs are mostly derived from protein-coding genes, being classified as exonic, intronic and exon-intron circRNAs, and from tRNA splicing reactions in a few cases. In prokaryotes, such as bacteria and archaea, circRNAs are derived from group I introns and tRNA and rRNA introns [84,87,88]. Findings about mitochondrial encoded circRNAs (mt-circRNAs) are still very sparse. The first evidence was in 1980–1983, which demonstrated that yeast mtGenome is able to naturally produce circRNAs. In these cases, mt-circRNAs are derived from mRNAs excised introns, but their functions remain unknown (Table 1) [65,66,67].

Later, a study demonstrated that introns of wheat mitochondrial genes produce circRNAs, suggesting the plasticity and tolerance of the splicing machinery in plants [68]. This was recently corroborated by evidence of mt-circRNAs in barley. In this case, exonic circRNAs are produced by mitochondrial genes involved in a wide range of metabolic pathways. Interestingly, these exonic circRNAs are present in barley leaves, grains and in grain transfer cells, and their expression varies in response to micronutrients [69].

In mammalians, there is only limited evidence of mt-circRNAs. Gao et al. (2018) found three mt-circRNAs by studying circRNAs expression in cattle testis, but their functions were not elucidated [70]. In mice, it was demonstrated that a nuclear circRNA called MFACR (mitochondrial fission and apoptosis-related circRNA) regulates mitochondrial fission and apoptosis in the heart by regulating miR-652-3p, suggesting that circRNAs are potential biomarkers of cardiovascular diseases [71].

In humans, a study evaluated the expression of circRNAs in subcellular localizations of HepG2 cells and found a wide distribution of circRNAs among nucleus, cytoplasm, ribosome, cytosol and exosome. They also found 118 mt-circRNAs with low abundance in mitochondria but didn’t provide more information and neither perform any further experimentation [72].

Considering that human mtGenome is smaller and lacks introns, human mitochondria machinery must use another mechanism for circRNAs biogenesis rather than intron lariats observed in yeast and plants. It is possible that a similar mechanism for chimeric lncRNAs production is applied to circRNAs in order to provide more plasticity to the non-canonical splicing events in human mitochondria.

### 7.3. miRNAs

Among the small ncRNAs subclasses, miRNAs are the most well-known. They are an important class of post-transcriptional regulators of gene expression by targeting mRNAs. In mammals, mature miRNAs associate with Argonaute proteins (Ago2) to form a ribonucleoprotein complex named RNA-induced silencing complex (RISC) and promote RNA interference (RNAi) [89].

The first comprehensive evidence of the presence of miRNAs in mitochondria was described in 2009 (Table 1). The authors found 15 nuclear-encoded miRNAs in mitochondria isolated from normal adult rat liver. Functional enrichment suggested the involvement of these miRNAs in apoptosis, cell death, and cell division. Interestingly, highly abundant miRNAs in liver, such as let-7a and miR-21, were absent in these isolated mitochondria, suggesting the existence of a specific population of miRNAs in the organelle [73].

In a similar experiment, Bian et al. (2010) found unique miRNAs enriched in mitochondria isolated from mouse liver in comparison to mouse liver tissue. The 20 miRNAs with the highest signals in mitochondria may be involved in the control of mitochondria gene expression and other general cellular processes such as apoptosis, proliferation and differentiation [74].

Later, it was found 57 differentially expressed miRNAs between mitochondria and cytosol in HeLa cells, suggesting that there is a subpopulation of miRNAs that are compartmentalized especially in mitochondria. Thirteen of these miRNAs were predicted as regulators of the mitochondrial genome and were termed as mitomiRs because they map to mitochondrial genes or to mitochondria-related nuclear genes and pseudogenes [75]. Therefore, the term mitomiR refers to miRNAs localized in mitochondria whether transcribed from the nuclear or from the mitochondrial genome [47].

The authors that firstly described the mitochondrial transcriptome also found 31 small ncRNAs (21 nt and 26 nt size classes) derived from 17 mitochondrial loci, being most of them produced from tRNA genes (86%), followed by protein-coding genes (12%) and rRNA genes (2%). These mt-ncRNAs presented a large and dynamic range of expression within different cell types, but their biological roles are still unknown [46].

In a similar study, Ro et al. (2013) identified thousands of small ncRNAs produced by the murine and human mtGenomes, being most of them derived from sense transcripts of the host mitochondrial genes. The authors also demonstrated the absence of RNAi machinery (Ago2 and Dicer) inside mitochondria and showed that Dicer inactivation did not abolish these miRNAs’ expression. These two findings suggested that there is an alternative RNAi-like machinery operating inside the organelle [76].

Interestingly, Barrey et al. (2011) searched *in silico* for miRNAs candidates in the mtDNA and found 33 human pre-miRNAs and 25 miRNAs. Three of them (pre-mir-302a, pre-let-7b and mir-365) were validated and described as located in mitochondria of human myoblasts. The authors also investigated the expression of 742 human miRNAs in mitochondria by RT-qPCR and found expression of 46 of them, which presented 80 predicted target sites in the mtGenome, suggesting their role in silencing of mitochondrial mRNAs [77].

In a different study, several miRNAs were found highly expressed in the mitochondrial fraction when compared to heart cells in rats. Among them, miR-181c (nuclear-ncRNA) was demonstrated to regulate COX1 by inhibiting its translation instead of degrading it, suggesting that this miRNA regulates mitochondrial functions [78].

Sripada et al. (2012) evaluated the global expression of small ncRNAs in mitochondria and found 428 already known and 196 putative novel miRNAs in HEK293, and 327 already known and 13 putative novel miRNAs in HeLa cells. Of these, only four aligned to mtGenome (hsa-miR-4461, hsa-miR-4463, hsa-miR-4484 and hsa-miR-4485). Overall, gene ontology analysis showed that these miRNAs are associated with RNA turnover, apoptosis, cell cycle and nucleotide metabolism [79].

Curiously, 78 miRNAs were found differentially expressed between two mitochondrial subpopulations after type 1 diabetic insult of rat heart tissues. This finding demonstrates that the miRNAs are differentially distributed in spatially distinct mitochondrial subpopulations of a tissue, evidencing their high grade of specificity. Among the 78 found miRNAs, miR-378 was shown to regulate the mitochondrial gene *ATP6* and its overexpression is potentially involved in decreasing activity of ATP synthase [80].

As mentioned before, even though miRNAs were found in mitochondria, this organelle seems to lack the canonical miRNAs biogenesis machinery [76]. Nuclear-encoded miRNAs are translocated into the mitochondria after being processed in the cytosol by the conventional pathway using nuclear and cytosolic enzymes [78,80]. On the other hand, it was demonstrated that inactivation of mitochondria leads to a strong decrease of miRNA-mediated RNAi efficiency by causing delocalization of endogenous Ago2 from P-bodies and affecting an early step of RISC assembly. This means that mitochondria may serve as a reservoir of not only miRNAs but also of ATP for RISC assembly in the P-bodies. Therefore, it seems that mitochondria may have an amphoteric role in RNAi pathways, serving as both target and regulator [90,91].

It is important to mention that the relation between mitochondria and miRNAs is even wider. In addition to mitomiRs, there are several miRNAs that indirectly modulate the mitochondrial activities, including energy metabolism, mitochondrial dynamics (fusion and fission) and apoptosis; they have also been associated with mitochondrial-related diseases such as type 2 diabetes, Parkinson’s disease and cancer [10,47,92].

### 7.4. piRNAs

Piwi-interacting RNAs are a class of small ncRNAs still poorly understood, but they became known as genome guardians due to their protection of the genome by promoting transcriptional and post-transcriptional silencing of transposable elements via DNA methylation and RNAi, respectively. To execute their functions, piRNAs are associated with PIWI proteins (Ago3, piwi and Aub), which are a subfamily of Argonaute proteins as well as Ago2 [93,94].

Initially, silencing of transposable elements was thought to be restricted to germline cells, in which dysfunction of this mechanism would cause genomic instability that could be passed on to the following generations – a transgenerational inheritance. However, recent studies have also demonstrated this mechanism in somatic tissues, which has been described as a possible cause for some diseases, including cancer [94].

So far, only two studies evaluated the presence of piRNA transcripts in mitochondria. Sripada et al. (2012) were the first to describe it – they found a unique population of small ncRNAs in mitochondria, including miRNAs, piRNAs and other types of non-epigenetic small ncRNAs [79]. Kwon et al. (2014) analyzed human normal and cancer cell lines and found 29 piRNAs that formed perfect matches with mtDNA, being most of them derived from tRNAs genes. These results strongly suggest that processing of piRNA precursors into mature piRNAs occurs inside the organelle, although the mature isoform can also be found in cytoplasm and nucleus [81].

**Figure 3:**
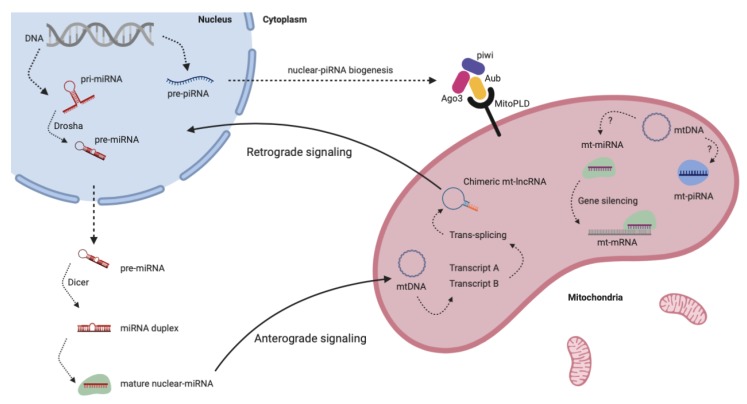
Mitochondria and non-coding RNAs. Scheme showing the complex communication between nucleus and mitochondria through ncRNAs. Nuclear-ncRNAs (miRNAs, for instance) regulate mitochondrial gene expression by anterograde signaling, while mt-ncRNAs (such as lncRNAs) regulate nuclear functions by retrograde signaling. Also, mitochondria produce its own piwi-interacting RNAs (piRNAs) and participate in nuclear-piRNAs’ biogenesis.

Additionally, it is important to emphasize that mitochondria have been described as essential to piRNAs biogenesis. In many species, piRNA biogenesis occurs near mitochondrial surface and involves mitochondrial membrane factors, such as MitoPLD in mammals and Zucchini (Zuc) in *Drosophila* (Figure 3). Some studies showed that mice and flies lacking MitoPLD/Zuc, respectively, have decreased piRNA biogenesis and maturation, leading to upregulation of retrotransposons [95,96,97]. Therefore, mitochondria seem to act as a scaffold for piRNA processing machinery, but the exact interplay of all involved factors is still poorly understood [98].

## 8. Non-coding RNAs and Mitochondrial Diseases

Mitochondrial diseases, such as MELAS (Mitochondrial Encephalopathy, Lactic Acidosis, and Stroke-like episodes) and MERRF (Myoclonic epilepsy with ragged- red fibers), are related to a maternal inheritance and are canonically caused by genomic mutations in mt-tRNAs. However, it has been shown that MELAS phenotype can also occur as a consequence of an abnormal regulation of miRNAs [99,100]. In MELAS, the canonical mt-tRNAs mutations prevent modifications of the anticodon wobble U34, affecting their function in translation [101]. These U34 modifications depend on the nuclear proteins GTPBP3, MTO1 and TRMU, which can be regulated by the expression of miR-9 and miR-9*. Abnormal expression of miR-9/* can lead to the downregulation of those proteins, contributing to a MELAS phenotype, even in the non-mutated mitochondria [99,100].

This is the only report associating ncRNAs to mitochondrial diseases per se. Other studies have shown the involvement of ncRNAs in various mitochondrial-related diseases, such as neurodegenerative diseases, diabetes and cancer. It is important to note that most of these studies focus on nuclear-ncRNAs regulating mitochondrial functions, and not on mt-ncRNAs [90].

It is widely accepted that cancer cells change their metabolic phenotype by enhancing glycolysis and reducing OXPHOS in a process referred to as Warburg effect. Under hypoxic conditions, this process is activated by HIF-1α and provide growth advantages for tumors. A previous report showed that lincRNA-p21, a nuclear-encoded long intergenic non-coding RNA (linc), is able to promote HIF-1α accumulation, leading to glycolysis under hypoxia. The feedback loop between HIF-1α and lincRNA-p21 was validated in mouse xenograft models, indicating this lincRNA as a therapeutic target for cancer [102]. This is just an example of how nuclear-ncRNAs are associated to mitochondrial-related diseases.

To date, only a few studies have raised the possibility of involvement of mt-ncRNAs in human diseases. Most of these studies were cited in the section “Non-coding RNAs in mitochondria” of this paper. For instance, SncmtRNA-1, SncmtRNA-2, ASncmtRNA-1 and ASncmtRNA-2 were associated with cancer [53,54,55,58], while some mt-lncRNAs and mt-circRNAs were reported in association to cardiovascular diseases [59,71].

Overall, the association between mt-ncRNAs and human diseases must be strong as they are crucial for cellular homeostasis and intracellular communication. Their involvement in these processes make them potential biomarkers for several diseases, evidencing the emergent need for functional and applied studies in mitochondrial epigenetics.

## 9. Perspectives

Epigenetic factors have been increasingly applied in the translational and clinical routine as therapy for many diseases. Currently, there are several epigenetic drugs commercially available and some in clinical trials, being all of them developed based on the nuclear epigenetics.

In this article, we provided enough information to evidence the wide importance of the mitochondria in the epigenetic field, indicating its therapeutic potential. However, the translation of mitochondrial epigenetics to clinical routine is still far from reality due to the lack of information and functional studies.

Our knowledge on the involvement of mitochondrial epigenetics in intrinsic functions of these organelles (such as OXPHOS and apoptosis) is still very limited, and it is even smaller when external and indirect cellular functions are considered. For instance, mitochondria have been related to ageing, which occurs, in part, in response to environmental factors, and mt-ncRNAs may be involved in this process. Hence, it is widely accepted that the epigenetic mechanisms respond to environmental factors, but the impact of these factors in mitochondrial epigenetics remains unknown. It is possible that, in response to external factors, an epigenetic imbalance in mitochondria may affect their communication to the nucleus, causing damages in the fine-tune cellular regulation.

Additionally, there are some points that have not yet been addressed, including the functional role of chemical modifications in TFAM, the presence of histones inside the mitochondria and mt-ncRNAs’ biogenesis and turnover.

Molecular mechanisms underlying trafficking of ncRNAs between nucleus and mitochondria are only now emerging. Mutations in these transport proteins (such as HuR and GRSF1) may cause damages in several cellular functions in which these ncRNAs are involved. The measurement of its impact is even more complicated considering the different grades of heteroplasmy that a cell may present and its threshold effect.

Furthermore, there is no data about the export of mt-ncRNAs to the extracellular environment into microvesicles and exosomes. This mechanism is important for the horizontal transfer of ncRNAs between cells and it is possible that both mt-ncRNAs and nuclear-ncRNAs may be involved in remote communication and activation of signaling pathways. Besides the clinical field, mitochondrial epigenetics may also be applied to evolutionary studies. In this regard, considering the inheritable features of the epigenetic mechanisms and that the environmentally induced phenotypes may persist for several generations (e.g., transgenerational inheritance), mitochondrial epigenetics might also explain many aspects of evolution, as well as being applied as marks in natural populations.

## 10. Conclusions

Mitochondria are essential for cellular homeostasis, being responsible for several biological processes, including OXPHOS and apoptosis. Surprisingly, if we consider the epigenetic scenario, mitochondria are even more important than previously thought to be and their influence goes far beyond the concept of “powerhouses of the cell”.

Mitochondria have a categorical involvement in cellular epigenetic mechanisms, functioning as both target and source of these processes. Indirectly, mitochondria participate of all DNA methylation and histones modification processes by producing the main chemical molecules used in these mechanisms and by participating of piRNAs’ biogenesis. Additionally, mitochondrial gene expression is regulated by mtDNA methylation and by intrinsic and extrinsic epigenetic factors (mt-ncRNAs and nuclear-ncRNAs, respectively).

Nucleus regulates mitochondrial functions not only by producing OXPHOS-related proteins but also at an epigenetic layer by RNAi pathways that manipulate mitochondrial proteome, evidencing that anterograde signals are crucial in all functional aspects. Disturbance of these signals seems to be severely related to some diseases and healthy conditions and it should be better understood.

The crosstalk between nucleus and mitochondria is deeply regulated by several synergistic factors and ncRNAs represent a novel layer in this intense regulation network. Overall, this article reinforced the importance of mitochondria in different aspects and evidenced that epigenetics is a brand new mitochondrial-related perspective that needs to be further explored.

## Figures and Tables

**Table 1:** Characterization of nuclear-ncRNAs and mt-ncRNAs and their related diseases and functions.

ncRNAs in Mitochondria	Type	Genomic Origin	Probable Signaling	Function	Related Diseases	Organism	Reference
1 lncRNA	Chimeric	Mitochondrial	Retrograde	NS	NA	*Mus musculus*	[48]
3 lncRNAs	Simple	Mitochondrial	Retrograde	NS	Cervical cancer	*Homo sapiens*	[49]
1 lncRNA	Chimeric	Mitochondrial	Retrograde	NS	NA	*Mus musculus*	[50]
1 lncRNA	Chimeric	Mitochondrial	Retrograde	NS	NA	*Mus musculus* and *Homo sapiens*	[51]
1 lncRNA	Chimeric	Mitochondrial	Retrograde	Cell proliferation	Multiple cancers	Homo sapiens	[52]
2 lncRNAs	Chimeric	Mitochondrial	Retrograde	Cell cycle regulation	Multiple cancers	Homo sapiens	[53]
2 lncRNAs	Chimeric	Mitochondrial	Retrograde	Cell survival	Renal cell carcinoma	Homo sapiens	[54]
2 lncRNAs	Chimeric	Mitochondrial	Retrograde	Apoptosis	Multiple cancers	Homo sapiens	[55]
2 lncRNAs	Chimeric	Mitochondrial	Retrograde	NS	Melanoma	*Mus musculus* and *Homo sapiens*	[56]
1 lncRNA	Chimeric	Mitochondrial	Retrograde	Cell cycle regulation	NA	*Mus musculus* and *Homo sapiens*	[57]
2 lncRNAs	Chimeric	Mitochondrial	Retrograde	Cell proliferation	HPV infection and cervical cancer	Homo sapiens	[58]
9 lncRNAs	NS	Mitochondrial	Retrograde	NS	Heart failure	Homo sapiens	[59]
1 lncRNAs	NA	Nuclear	Anterograde	Mitochondrial DNA replication	Cervical cancer	Homo sapiens	[60]
1 lncRNA	NA	Nuclear	Anterograde	NS	NA	Mus musculus	[61]
1 lncRNA	NA	Nuclear	Anterograde	Estrogen transduction signaling	Multiple cancers	Homo sapiens	[62]
1 lncRNA	NA	Nuclear	Anterograde	mt-tRNA maturation	Cervical cancer	Homo sapiens	[63]
1 lncRNA	NA	Nuclear	Anterograde	RNA transport	NA	Yeast and mammalian	[64]
1 circRNA	Intronic	Mitochondrial	Retrograde	NS	NA	Saccharomyces cerevisiae	[65]
2 circRNAs	Intronic	Mitochondrial	Retrograde	NS	NA	Saccharomyces cerevisiae	[66]
circRNAs*	Intronic	Mitochondrial	Retrograde	NS	NA	Saccharomyces cerevisiae	[67]
circRNAs*	Intronic	Mitochondrial	Retrograde	NS	NA	Triticum aestivum	[68]
62 circRNAs	Exonic	Mitochondrial	Retrograde	Micronutrient response	NA	Hordeum vulgare	[69]
3 circRNAs	NS	Mitochondrial	Retrograde	NS	NA	Bos taurus	[70]
1 circRNA	Exonic	Nuclear	Anterograde	Regulation of apoptosis and mitochondrial fission	Cardiovascular diseases	Mus musculus	[71]
118 circRNAs	NS	Mitochondrial	Retrograde	NS	Liver cancer	Homo sapiens	[72]
31 sncRNAs	NA	Mitochondrial	Retrograde	NS	Osteosarcoma	Homo sapiens	[46]
15 miRNAs	NA	Nuclear	Anterograde	Apoptosis, cell proliferation and differentiation	NA	Mus musculus	[73]
40 miRNAs	NA	Nuclear	Anterograde	Apoptosis, cell proliferation and differentiation	NA	Mus musculus	[74]
57 miRNAs	NA	54 nuclear	Anterograde	Cell cycle, ATP synthesis and mitochondrial translation	Cervical cancer	*Homo sapiens*	[75]
	NA	3 mitochondrial	Retrograde				
1499 sncRNAs	NA	Mitochondrial	Retrograde	Control of mitochondrial gene expression	NA	*Mus musculus*	[76]
2540 sncRNAs	NA		Retrograde			*Homo sapiens*	
25 miRNAs	NA	Mitochondrial	Retrograde	Silencing of mitochondrial mRNAs	NA	*Homo sapiens*	[77]
46 miRNAs	NA	Nuclear	Anterograde				
15 miRNAs	NA	Nuclear	Anterograde	Regulate mitochondrial functions	NA	*Mus musculus*	[78]
428 miRNAs (HEK293)	NA	Nuclear	Anterograde	Apoptosis, cell cycle and nucleotide metabolism	NA	*Homo sapiens*	[79]
327 miRNAs (HeLa)	NA	Nuclear	Anterograde		Cervical cancer		
4 miRNAs (HEK293/HeLa)	NA	Mitochondrial	Retrograde		NA/Cervical cancer		
piRNAs (HEK293/HeLa)*	NA	Mitochondrial	Retrograde	NS	NA/Cervical cancer		
78 miRNAs	NA	Nuclear	Anterograde	Silencing of mitochondrial mRNAs	Type 1 Diabetes Mellitus	*Mus musculus*	[80]
29 piRNAs	NA	Mitochondrial	Retrograde	NS	Multiple cancers	*Homo sapiens*	[81]

NS: Not Studied; NA: Not Applicable; *: Unspecified number.

## Abbreviations

OXPHOS	oxidative phosphorylation
mtGenome	mitochondrial genome
mtDNA	mitochondrial DNA
nDNA	nuclear DNA
ncRNAs	non-coding RNAs
ETC	electron transport chain
ROS	reactive oxygen species
ADP	adenosine diphosphate
ATP	adenosine triphosphate
MOMP	mitochondrial outer membrane permeabilization
TFAM	transcription and mtDNA maintenance factor
HIF1	hypoxia inducible factor 1
SIRT	sirtuins
tRNAs	transfer RNAs
rRNAs	ribosomal RNAs
D-loop	displacement loop
DNMTs	DNA-methyltransferases
CpG sites	cytosine-guanine dinucleotides
lncRNAs	linear long non-coding RNAs
circRNAs	circular RNAs
miRNAs	microRNA
piRNAs	piwi-interacting RNAs
nuclear-ncRNAs	nuclear-encoded non-coding RNAs
mt-ncRNAs	mitochondrial encoded non-coding RNAs
HuR	human anti-gen R
GRSF1	G-rich RNA sequence-binding factor 1
RNase MRP	RNA processing endoribonuclease
SRA	steroid receptor RNA
RBP	RNA binding protein
Ago2	Argonaute
RISC	RNA-induced silencing complex
RNAi	RNA interference
Aub	aubergine
Zuc	zucchini
MELAS	Mitochondrial encephalopathy, lactic acidosis, and stroke-like episodes
MERRF	myoclonic epilepsy with ragged-red fibers
lincRNA	long intergenic non-coding RNAs
